# Geolocators Reveal Migration and Pre-Breeding Behaviour of the Critically Endangered Balearic Shearwater *Puffinus mauretanicus*

**DOI:** 10.1371/journal.pone.0033753

**Published:** 2012-03-21

**Authors:** Tim Guilford, Russell Wynn, Miguel McMinn, Ana Rodríguez, Annette Fayet, Lou Maurice, Alice Jones, Rhiannon Meier

**Affiliations:** 1 Department of Zoology, University of Oxford, Oxford, Oxfordshire, United Kingdom; 2 Marine Geoscience Group, National Oceanographic Centre, Southampton, Hampshire, United Kingdom; 3 Conservation Department, Skua Gabinete de Estudios Ambientales Sociedad Limitada Profesional, Palma de Mallorca, Balearic Islands, Spain; 4 British Geological Survey, Natural Environment Research Council, Crowmarsh Gifford, Oxfordshire, United Kingdom; University of Western Ontario, Canada

## Abstract

Using combined miniature archival light and salt-water immersion loggers, we characterise the year-round individual at-sea movements of Europe's only critically endangered seabird, the Balearic shearwater *Puffinus mauretanicus*, for the first time. Focusing on the non-breeding period, we show that all of the 26 breeding birds tracked from their breeding site on Mallorca in the Mediterranean Sea successfully made a 2–4 month migration into the Atlantic Ocean, where they utilised well-defined core areas off Portuguese and French coasts. As well as identifying high-risk areas in the Atlantic, our results confirm that breeding birds spend most of the year concentrated around productive waters of the Iberian shelf in the western Mediterranean. Migration phenology appeared largely unrelated to the subsequent (distinctly synchronous) breeding attempt, suggesting that any carry-over effects were compensated for during a long pre-laying period spent over winter in the Mediterranean. Using the light and salt-water immersion data alone we were also able to characterise the pattern of pre-laying visits to the colony in considerable detail, demonstrating that breeding pairs appear to coordinate their over-day visits using a high frequency of night-time visits throughout the winter. Our study shows that geolocation technology is a valuable tool for assessing the spatial distribution of risks to this critically endangered species, and also provides a low-impact method for remotely observing the detailed behaviour of seabird species that may be sensitive to disturbance from traditional study methods.

## Introduction

Understanding the behaviour of migratory species that are endangered can be especially problematic because of their rarity and their sensitivity to disturbance, yet migration is a critical stage of their life history. A striking example is the Balearic shearwater *Puffinus mauretanicus*, a member of the highly pelagic Procellariiforms and currently Europe's only critically endangered seabird species [1]. The known breeding population comprises just ∼3200 pairs [2], with models suggesting that adult survival at sea may be sufficiently low to precipitate extinction within a few generations [3]. Whilst recent studies have contributed valuable information on the species' life history and dietary requirements [4]–[7], remarkably little is known about its at-sea behaviour, especially the post-breeding migration from its breeding sites which are restricted to the Balearic islands in the western Mediterranean [2]. At-sea sightings suggest that birds migrate into the Atlantic in the late northern summer, occupying relatively shallow, coastal waters along northeast Atlantic coasts where they may both exploit, and become vulnerable to by-catch from, human fishing activity [8], [9]. In addition to the inter-annual volatility of populations of prey fish, such as the small pelagic sardine [9], other factors such as longer-term climate change [10] and changes in discard availability [11] may influence the at-sea distribution of this species. In particular, there has been a large increase in numbers of birds recorded off northwest France and southwest UK since the mid-1990's [10], [12]–[15], with recent aggregations off northwest Brittany holding as many as 6000 birds [16]. As with all such sightings, however, the provenance and age of birds forming these aggregations are unknown.

Here we report the first successful attempt, using miniature geolocation technology, to study directly the individual migratory movements of Balearic shearwaters of known provenance and breeding status across the entire annual cycle. We show that breeding birds from the species' largest known cave colony exhibit a consistent migration immediately post-breeding (no bird failed to migrate), concentrating in two areas off western Portugal and western France. None of the tracked breeding birds penetrated north of the Bay of Biscay in summer 2010, despite ∼25% of the World population being concentrated off northwest France at this time. This implies colony- or age-specific migration strategies may be employed by this species. Furthermore, we demonstrate that data from geolocators can be used to characterise the migratory period rather precisely, and relate this to breeding phenology and success in a way that provides detailed behavioural information during breeding without disturbance to birds at the colony. In addition, by comparing this behaviour in the two birds within each nesting pair it is also possible to sex individuals uninvasively. We suggest that archival light-logging technology may offer a powerful tool for studying the behaviour of sensitive species with minimum disturbance.

## Methods

### Ethics Statement

All work was conducted in accordance with the appropriate Balearic Government (Servei de Protecció de Espècies) guidelines, and under permit numbers CAP31/2011 and CAP04/2010 from the Balearic Government (Servei de Protecció de Espècies).

The study was conducted at the largest extant breeding colony of Balearic shearwaters currently known, Sa Cella cave on the northwest coast of Mallorca, where a subset of nesting sites, usually scrapes on sediment surfaces or under protected shelves, have been numbered and monitored for some years [6], [17] allowing us to utilise pairs of ringed birds of known established breeding success. To reduce impact, visits to nesting areas of the cave were minimized, and conducted using only dim red light or a night-vision scope. Birds were captured on the nest by hand and placed into cloth bags for weighing, carriage and handling. Instrument deployment and downloading or removal took place in dim light close to the cave entrance. BAS Mk15 geolocators weighing 2.4 g were attached to 17 breeding pairs (34 individuals) by two small cable ties to a hand-fashioned Darvik ring custom-sized to an incubating bird's leg during early April 2010. Correction fluid was used to mark temporarily the head and back after deployment to enable remote identification of changeovers at each nest. During late March 2011 visits were made to the cave to recover geolocator data, which were downloaded *in situ* whilst the logger remained on the leg to allow data-gathering in future years. At the same time, birds' legs were examined for signs of damage and faulty loggers or worn Darvik rings were replaced. Breeding success in the year of deployment was estimated during routine monitoring visits later in the season, as was that of 27 control nests interspersed amongst the experimental nests to provide an even match of conditions as far as possible.

Light-level data were analysed in BasTrak software to provide approximate positions twice daily throughout the year, based on day/night transitions estimated using a standard light threshold of 10, linear interpolation, and filtering of apparent light or dark period of less than 4 hours. For a sub-set of 10 birds, a sunset angle was chosen individually for each logger/bird combination which localized positions close to the colony on days either side of periods during which birds' positions could be surmised to be in the colony because of sustained logged daytime darkness (for example, during incubation). Most datasets were best ground truthed with an angle of −3.5, so this was then chosen for all remaining data sets. Resultant position estimates were then filtered by eye to remove locations requiring movement of more than 2 degrees, latitude or longitude, in 24 hours.

Because passage through the Straits of Gibraltar involves a purely longitudinal progression (passage is latitudinally constrained by Iberia to the north, and Africa to the south), it is possible to define individual Mediterranean exit and re-entry dates precisely using geolocator data despite inherent latitudinal inaccuracy, even around the equinox. Furthermore, the occurrence of complete daytime darkness in the logger trace allows identification of days spent in the colony, whilst sustained periods of night-time dryness in the salt-water immersion traces allows identification of visits to the cave during the night. We used these additional pieces of information from the loggers to pinpoint the phenology of annual events (migration, colony returns, egg laying) much more precisely than is possible using geolocation data alone.

## Results

### Impacts

Of the 34 deployments in 2010, 28 birds were recaptured breeding again at the colony in 2011, mostly in the same nests and with the same partners. All pairs hatched young successfully in 2011 post-deployment, but one chick failed to fledge. 6 birds were not re-encountered, giving a minimum annual survival of 82%. Although we did not measure over-winter return in the control nests, estimated adult survival between 1998–2002 was similar at 78% [3]. All but three experimental pairs bred successfully in the year of deployment, with two eggs failing to hatch and one chick failing to fledge. Hatching success was as good as that in controls nests (0.88 vs 0.82, Fisher's Exact test *p* = 0.271), as was fledging success (0.70 vs 0.63, Fisher's Exact test *p* = 0.198). One bird returned missing its geolocator, and one geolocator failed to yield data, leaving 26 complete data sets.

### Sexing behaviourally

We were able to sex birds behaviourally. Petrels in general, and the closely related Manx shearwater (*Puffinus puffinus*) in particular [18], show a distinct pre-laying exodus in which the female is absent from the nest for 2–3 weeks whilst she builds her egg, yet the male continues to revisit the nest on most nights. We used the pattern of salt-water immersion data to identify night-time visits to the colony (see below). In the 10 pairs for which we have both birds tracked, one individual was always absent from the colony for an extended period prior to the start of incubation (the pre-laying exodus) whilst the other individual was apparently present on most nights. We used this distinction to assign sex, and durations of pre-laying absence from the colony were non-overlapping between putative males and females (male absence, median 1.5 days, range 0–4 days; female absence: median 13.5 days, range 5–22 days). We then cross-verified this categorization by identifying which bird provided the first incubation stint (which should be the male: 18). On 9 out of 10 occasions this cross-check was clear cut and correct, whilst on one occasion there was a slight ambiguity caused by a short (one day) first incubation stint. We then applied the pre-laying absence criterion to sex the remaining 6 birds.

### At-sea movements

**Figure 1:**
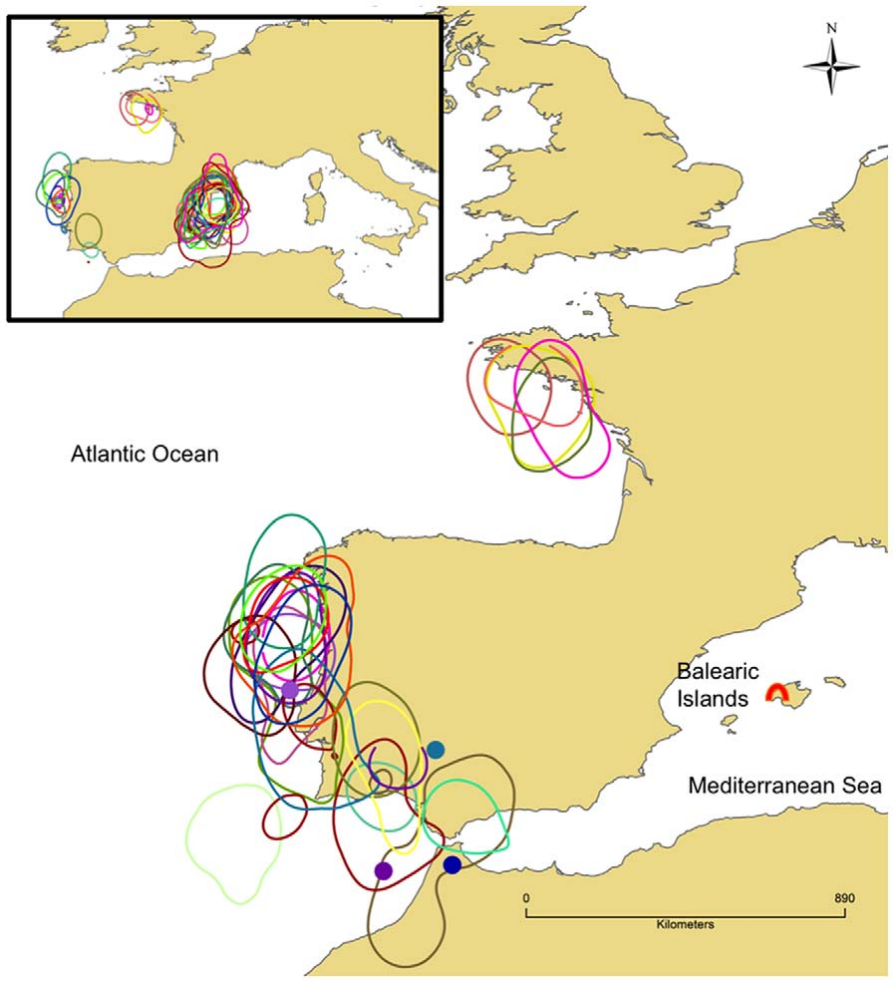
Balearic shearwater movements at sea. The inset shows the 50% occupancy kernels for all birds of all valid locations through the entire annual cycle, with each individual coloured separately. The main figure shows the 50% occupancy kernels for all birds of locations between leaving and returning to the Mediterranean on migration, in the same colours. The coloured circles are spatial median positions for four birds that made a second trip into the Atlantic post-migration (same individual colours). These latter estimated positions are very approximate, and should not be taken to signify that birds are inland. The red symbol is the position of the breeding colony at Sa Cella cave on Mallorca.

Figure 1 (inset) shows the 50% occupancy kernels for each individual's year-round daily locations, with each individual coloured separately. The data demonstrate clearly that birds divide their time principally between the breeding area centred north west of the colony on the Iberian shelf, where there is remarkable inter-individual consistency, and migration areas in the northeast Atlantic.

A clearer picture of the migration is obtained by filtering the data to exclude locations outside the migration period. Figure 1 (main figure) shows the 50% occupancy contours for all valid locations for each individual during migration alone, characterised as the period between first exit from and first re-entry to the Mediterranean. This demonstrates that all 26 tracked birds moved into the northeast Atlantic on post-breeding migration, returning to the colony in the autumn.

### Migratory phenology

**Figure 2:**
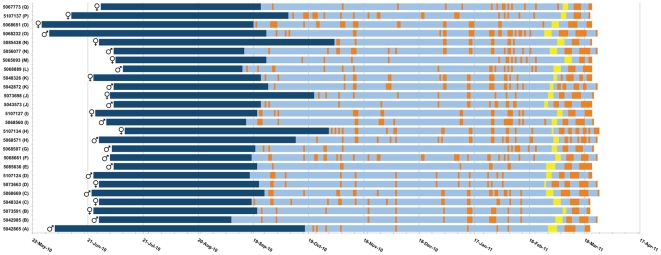
Individual pre-breeding phenologies. Individual phenology traces are represented for all 26 birds labeled by ring number, grouped by their original nest pairings (bracketed letters). Behaviourally determined sex is given for each bird. The first portion of each trace (dark blue) shows the timing and duration of migration, bounded by date first day in the Atlantic post-breeding and date of first return to the Mediterranean. Orange bars represent presumed over-day visits to the colony. The first incubation stint for each bird is shown in yellow, and the following asynchronous visits within pairs clearly show the pattern of coordinated incubation. Traces stop when devices were downloaded.

Figure 2 shows the individual timing of key events during the annual cycle determined using longitudinal progression or logged day-time darkness. Where both birds of an original pair were successfully tracked, they are grouped together to enable visualization of synchrony between pair members.

**Figure 3:**
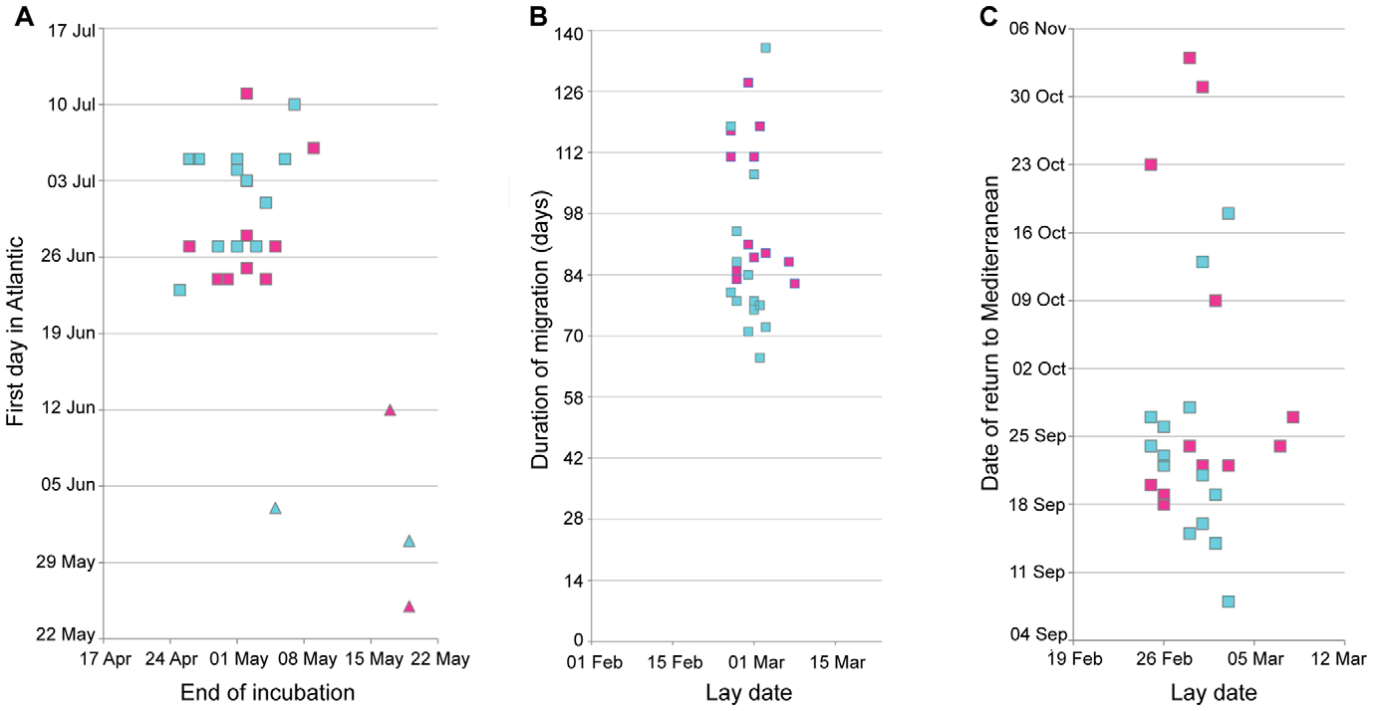
Relationships between timing of breeding and migration. **A**) Start of migration is not significantly related to last day of preceding incubation, except for failed breeders: males (blue); females (pink); successful breeders (squares); failed breeders (triangles). In neither males (blue) nor females (pink) does the duration of migration (**B**), or the date of return from migration (**C**), influence subsequent timing of breeding (plotted as lay date).

The timing of the Atlantic migration, its duration, and the timing of return to the Mediterranean were rather variable. Median date of passage into the Atlantic was 27/06/2010 (range 31/05/2010-11-07/2010). We were able to relate migration to the previous (2010) breeding attempt by identifying the apparent end of incubation from the pattern of logged day-time darkness. Those individuals whose breeding failed, stopped incubation latest probably because they were unwittingly sitting on infertile or failed eggs, and then left earliest on migration (shown in Figure 3A). Otherwise, the timing of hatching was not significantly related to the timing of the parents' subsequent migration (Spearman's rho = 0.298 for males (*p* = 0.347) and 0.216 for females (*p* = 0.549), excluding failed breeders).

The Atlantic migration lasted about 3 months (median 88 days; range 66–137 days), with failed breeders tending to spend the longest away from the Mediterranean (median duration 120 days for failed breeders versus 86 days for successful breeders; Mann-Whitney U test Z = 2.914, *p* = 0.004). There was no obvious relationship between the migratory destination and breeding failure, with two failed breeders travelling to southwest Brittany (females), and two to Portugal (males). However, migratory destination was related to sex, with all five birds travelling the furthest, to southwest Brittany, being females (Fisher's Exact test *p* = 0.012). Females spent longer on migration than males (median duration 91 days for females and 83 days for males; Mann-Whitney U test Z = 2.13, *p* = 0.033), even if failed breeders are excluded (median duration 90 days for females and 80 days for males; Mann-Whitney U test, Z = 2.605, *p* = 0.009). In fact, the females that did not travel as far as southwest France still spent longer on migration than males (Mann-Whitney U test Z = 1.986, *p* = 0.047, excluding all failed breeders), so the more distant destination was apparently not the cause of longer migration (there is no difference in duration of migration with destination amongst all females: Mann-Whitney U test Z = 1.467, *p* = 0.142).

Return from migration was also rather variable (median date 23/09/2010; range 08/09/2010–03/11/2010). All birds then spent a long pre-laying period during autumn and winter in the Mediterranean (predominantly) after return from migration (median 157 days, range 117–176 days), and this was the same for males (median 157 days) and females (median 159 days). Lay dates were estimated by the onset of incubation discerned in the pattern of logged day-time darkness, and especially clear in the abrupt pattern of asynchronous presence in the cave visible within pairs in Figure 2. In contrast to the events of migration, laying was remarkably synchronous, and was apparently unrelated to the length of migration (Figure 3B; Spearman's rho = 0.328 for males (*p* = 0.253) and 0.289 for females (*p* = 0.362)), or the date of first arrival back in the Mediterranean (Figure 3C; Spearman's rho = 0.323 for males (*p* = 0.260) and 0.005 for females (*p* = 0.987); see below) suggesting that there is no obvious carry-over effect from migration to the timing of breeding.

### Pre-laying behaviour

**Figure 4:**
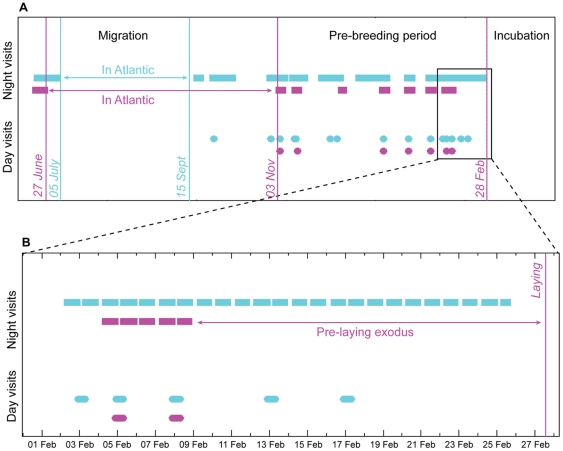
Pre-laying behaviour. Panel A shows the pattern of night-time visits (squares) and day-time visits (circles) for two birds in typical pair (N), the male in blue (556077) and the female in pink (5085438), over the entire post-migration, pre-laying period. Panel B shows a zoomed section covering the three week period before laying, which clearly shows the contrast between male visiting behaviour and female pre-laying exodus. Vertical lines show dates of exit into the Atlantic, entry into the Mediterranean, and laying, for both the male (blue) and the female (pink).

We used prolonged periods (>2 hrs) of continuous dryness in the salt-water immersion data to identify periods on land at night, presumed to be visits to the colony, during the pre-laying period. Birds spent many nights, or part thereof, at the colony between migration and laying (females, median 42 nights, range 22–62 nights; males, median 61.5 nights, range 46–81 nights), with males visiting the colony during this pre-laying period significantly more often than females (Two-sample t-test, t = −4.37, *N* = 26, *p*<0.001). Birds first arrived back at the colony within one or two days of entering the Mediterranean (median delay 2 days; range 0–18 days), usually spending a day in the colony shortly, sometimes immediately, afterwards (median delay after migration 3 days; range 0–19 days), and continued to revisit the colony over-day at intervals throughout the winter until breeding in the spring (see Figure 2). Individuals within a pair are highly synchronous in their pattern of over-day visits to the colony, with between 28% and 92% (median 59%) of days spent together. The pair with the lowest degree of synchrony (28% & 31% of visits together; birds 5068532 & 5068651) had a failed egg in 2010, and were the earliest to lay in 2011, suggesting a possible relationship between pair synchronization and breeding performance. We also noticed that two birds (5068661 & 5107137) originally breeding in different pairs in 2010 showed synchronous visits during the winter return, and these two birds, one of whose partner failed to return, were subsequently found breeding successfully together in 2011. Synchronous day-time visits to the colony were usually preceded by a series of night-time visits by one or other bird which continued until the second bird made a night-time return, to be followed immediately by a day together in the cave. This suggests that synchrony emerges as a consequence of the pair meeting at the colony. Figure 4 shows an example of the pattern of night-time and day-time visits for a typical pair, both throughout the pre-laying period (Fig. 4A) and in the immediate run-up to laying when the female's pre-laying exodus clearly contrasts with male night-time visiting behaviour (Fig. 4B).

**Figure 5:**
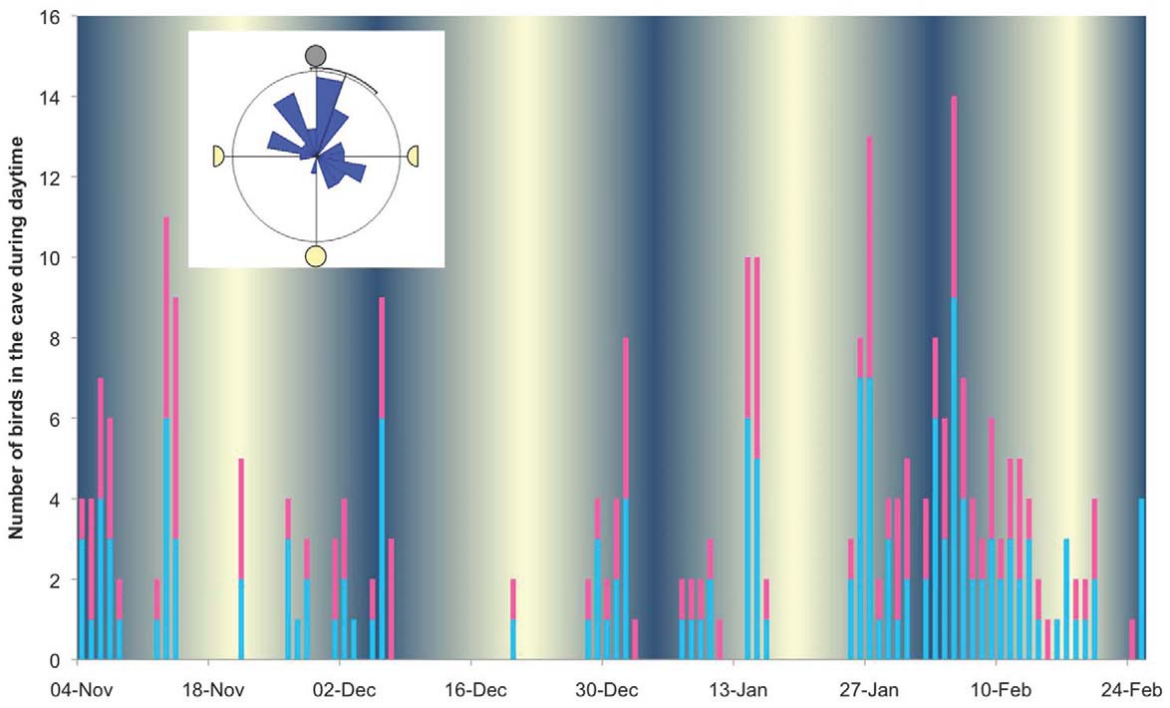
Distribution of day visits to colony in relation to lunar cycle. The main figure shows a histogram of over-day visits to the colony in relation to date and lunar cycle, for all 26 birds tracked and during the that portion of the pre-laying period in which all birds had returned from migration, but no bird had yet laid. Males are in blue, females are in pink. The Rose diagram (inset) shows the distribution of visits in relation to moon phase alone, with lunar progression clockwise, including one randomly selected bird from each of 10 pairs for which we have both partners' tracks, and 6 solo tracked birds (*N* = 16 birds, 89 day visits). Rayleigh test, R = 10.576, *p* = 2.55×10^−5^. V-test with new moon as expected mean, U = 4.324, *p* = 5.97×10^−6^. Circumferencial bar shows 95% confidence limits.

Over and above this within-pair synchrony, over-day visits to the colony were not randomly distributed, with visits, or periods of absence, clustered through the period (shown in figure 5). To eliminate the pseudo-replication effect caused by within-pair synchrony, we randomly excluded one bird from each pair for which we had both tracks, providing a sample of 10 to which we added the remaining 6 birds for whom we did not have the partner's track (Kolmogorov-Smirnov test: *N* = 16; ks2stat = 0.2105; *p*<0.011). There is a gradual build up of day-time activity in the cave towards breeding, with incubation preceded by a short period in which most birds, and all females, are absent (the pre-laying exodus). We related over-day visits to the colony to moon phase. We plotted in figure 5 (inset) the remaining 89 over-day visits on a circular diagram against the daily phase of the moon between end of migration and start of incubation. A Rayleigh test shows that visits are significantly non-randomly distributed (R = 10.576, *p* = 2.55×10^−5^), whilst a V-test with new moon as the expected mean shows that visits are significantly clustered towards the new moon (U = 4.324, *p* = 5.97×10^−6^) which is within the 95% confidence limits of the distribution.

Most birds spent the winter period between visits to the colony in the Mediterranean, but four birds showed an additional excursion into the Atlantic exiting the Mediterranean between 16^th^ and 25^th^ October for between 9 and 21 days. These excursions are plotted as spatial median positions in Figure 1, providing an approximate centre of location for the whole trip between entering and leaving the Atlantic. Because these involve rather few data, and probable rapid movement between different places which are spatially averaged, the positions are very approximate, especially in Latitude (for example, it is extremely unlikely that birds will actually be located over a landmass).

## Discussion

Geolocation technology is increasingly used to study the migration patterns of ever smaller or more sensitive pelagic seabird species [19]–[22], and at ever increasing scales [23]. Here we demonstrate its successful, low impact use to study migratory and pre-breeding behaviour in, and determine the sexes of, a critically endangered seabird species.

At least in so far as our one-year sample from the world's largest known breeding colony is representative, the breeding Balearic shearwater's year at sea is divided predominantly between two distinct phases. During spring breeding, and in the autumn and winter months beforehand (about three quarters of the year), movements are predominantly concentrated in a remarkably restricted area around the Balearic Islands where they breed, extending to the Spanish mainland coast and encompassing the productive shallow waters of the Iberian continental shelf. This finding is consistent with previous work, largely based on at-sea sightings [5], [24], [25], and it is here around the Iberian shelf where breeding birds are likely to be most at risk at sea from fisheries by-catch, pollution, or, potentially, renewable energy developments. We also resolve occasional westerly movements during this period, in some cases as far as the Atlantic. Soon after breeding, birds migrate into the northeast Atlantic to coastal regions off Portugal and western France, where they spend about one quarter of the year.

We were able to characterize the timing of post-breeding migration quite precisely because it involves passage through the narrow straits of Gibraltar where movement is almost entirely longitudinal, circumventing the usual latitudinal inaccuracy of geolocation techniques around the equinoxes. It appears that all breeding individuals migrate into the Atlantic from the Mediterranean soon after they have finished breeding in mid-summer (median 27^th^ June), presumably to exploit seasonally productive shelf waters around the Iberian and French Atlantic coasts, and to moult. Earlier attempts to track post-breeding dispersal, using satellite tracking [5], have suggested that at least some breeders may remain in the Mediterranean rather than migrate into the Atlantic, a result that contrasts with our own. This difference could be a genuine effect of differences between years, or perhaps an indication that the much heavier devices deployed in the earlier study disrupted normal behaviour. In our study, failed breeders make the Atlantic migration earliest, leaving before successful breeders have fledged their young, perhaps as soon as they perceive their own failure (as an egg that fails to hatch, for example). The Atlantic migration lasts around 3 months (median 88 days), but the longer migratory durations of failed breeders especially contribute to a rather variable length. Intriguingly, there is also a suggestion that females spend significantly more time in the Atlantic than males, but a larger sample will be needed to assess the generality of sex differences. Sightings of Balearic shearwaters apparently leaving the Mediterranean occur throughout the non-breeding period [5]. Our results show that an exodus of successful breeders is concentrated for a few weeks around the end of June, implying that the more broadly dispersed movements may be made up of young, immatures, and failed breeders. However, we also discovered that a small proportion of birds make (at least) an additional trip back into the Atlantic post-migration, a behaviour that presumably contributes to the sightings of outward movements throughout the non-breeding period.

Spatially, birds on migration appear largely to restrict their activity to one of two core areas off western Portugal or southwestern Brittany. Only one bird appeared to visit both areas. In our sample, only females travelled to southwestern Brittany raising the intriguing possibility that post-breeding migration strategies may differ, at least partially, between the sexes (echoing the differences in migratory duration discussed earlier). Previous observations have recorded concentrations of birds off these coasts before, and there may well be inter-annual shifts in distributions in relation, perhaps, to food availability [9], [12], [26]. Our results confirm that these areas, and the risks associated with them, are important for *breeding* individuals. However, and including the year that our study was conducted (2010), major concentrations of Balearic shearwater sightings have tended to be further north than these areas, off the coasts of northwest Brittany [16] and, increasingly frequently, off southwest UK [10], [15]. We found no evidence of Mallorcan breeding birds utilizing these areas, suggesting either that such sightings comprise predominantly non-breeding birds, or that birds breeding at different colonies may show migratory segregation. Numbers of sightings further north start to build up in late May before our successful breeders are on migration, and can peak in late September after many breeders have returned to the Mediterranean [13], [15], supporting the hypothesis that these are at least partially non-breeders. Intriguingly, an aggregation of approximately 6000 birds, representing as much as 25% of the estimated global population [2] was recorded off northwest Brittany in late July 2010 [16] at a time when all of our tracked Mallorcan breeders were further south. As well as age segregation, it is possible that migratory segregation between different colonies could also account for the lack of Mallorcan breeders in these concentrations. Migratory segregation between colonies could reduce the species' resistance to extinction, and determining the migratory patterns of birds breeding at other colonies in the Balearic Islands, such as Ibiza, is clearly important. In either case for approximately ¼ of the year, a large percentage of the world's population of breeding birds will be vulnerable to by-catch in these two core areas within the territorial waters of Portugal and France (a problem which could be worsened if the segregation we found between the sexes turns out to be robust in a larger sample).

Return from migration occurs predominantly during September (median 23/09/2010), and as early as 09/09/2010 in our study, but is quite variable. Using the presence of long dry periods in the salt-water immersion traces at night we were able to identify probable visits to the colony remotely from the geolocator logs alone. Strikingly, we found that most birds apparently visited the colony immediately (median 2 days) after returning to the Mediterranean, suggesting almost direct flight from the Atlantic. This calls into question earlier inferences from direct field observations that the presence of birds around colonies and on the nests in early September indicates that breeders may be present in the Mediterranean all year round [5]. Instead, it seems, breeding birds make a rapid return directly after migration in the Atlantic.

The period between migration and laying makes up close to half of the breeding shearwater's life-cycle (median 159 days for females, 157 days for males), when most of the time at sea is spent around the Iberian shelf. During this period, as previous observations have suspected [5], night-time visits to the colony are a frequent behaviour with males spending more than a third of nights apparently on land (median 39%), and females around a quarter (median 27%). Whilst nest defence may be one function of these night-time visits (particularly for males), the pattern strongly suggests a social function between pair members. Using the occurrence of logged day-time darkness we were also able to identify over-day visits to the colony, and relate these to the patterns of night-time visits. We found that birds appear to visit the colony on consecutive nights, returning to sea in the day, until their partner also visits at which point they are highly likely to spend the following day in the cave together. Such joint, over-day visits are generally followed by a more extended period at sea away from the colony. Synchronous over-day visits build up towards laying, culminating in a distinct pre-laying exodus period (a common feature of procellariiforms [18]) which allowed behavioural sexing remotely and uninvasively in this otherwise largely monotypic species. Over and above the within pair synchrony there is a significant relationship with the lunar cycle, visits significantly more likely to occur around the new moon. This might reflect a disadvantage to visiting the colony on bright nights (perhaps related to predation risk [18]), an advantage to foraging on bright nights, or an additional mechanism for synchronizing breeding across the whole colony. It is conceivable that weather effects are confounded with lunar phase in a single-year sample, and further data will be required to give more confidence in this result.

In contrast to the timing of events on migration, laying is highly synchronous, something which is strikingly found in some species of Procellariiform [27], [28], but not others [18]. It appears to be unrelated to duration of migration, or return date, suggesting that lay date itself is not subject to obvious carry-over effects. Lay dates appear to vary between colonies and years [5], so it is possible that within-colony synchrony is itself a strategy for reducing the local risk of egg or chick predation, and that the synchronous colony visiting behaviour that we find during the pre-laying period may function to facilitate this.

Although further study of inter-annual and inter-colony variation, and of different life stages, will be of crucial importance for properly informing conservation priorities, our results help to crystalize the risk structure to breeding individuals of this critically endangered species outside of the breeding season itself. Individuals spend around a quarter of their year, the late summer, on migration at coastal sites relatively localized off northeast Atlantic coasts, especially Portugal and France, and then return directly to their breeding sites to spend around 5 months visiting the colony rather frequently even before egg laying. Although excursions westwards, even back into the Atlantic, occur on some occasions, it appears from the frequency of visits to the colony, and the general location of core positions, that birds are already dependent on Iberian shelf waters long prior to breeding. Furthermore, pre-breeding colony visits may be important for synchronizing breeding, and may have other as-yet unknown functions, emphasizing that disturbance to colonies could be detrimental to breeding over a large part of the year. Combined archival light and salt-water immersion logging devices (geolocators) have proved to be a powerful, lightweight, and minimally invasive tool in this study, and may have considerable scope for understanding the movements and behaviour of other sensitive species if appropriately used.
